# A comparison between full-length 16S rRNA Oxford nanopore sequencing and Illumina V3-V4 16S rRNA sequencing in head and neck cancer tissues

**DOI:** 10.1007/s00203-024-03985-7

**Published:** 2024-05-07

**Authors:** Kenny Yeo, James Connell, George Bouras, Eric Smith, William Murphy, John-Charles Hodge, Suren Krishnan, Peter-John Wormald, Rowan Valentine, Alkis James Psaltis, Sarah Vreugde, Kevin Aaron Fenix

**Affiliations:** 1 Discipline of Surgery, Adelaide Medical School, The University of Adelaide, Adelaide, SA 5000 Australia; 2 Department of Surgery-Otolaryngology Head and Neck Surgery, The University of Adelaide and The Basil Hetzel Institute for Translational Health Research, Central Adelaide Local Health Network, Adelaide, SA 5000 Australia; 3 Department of Haematology and Oncology, Basil Hetzel Institute for Translational Health Research and The Queen Elizabeth Hospital, Central Adelaide Local Health Network, Adelaide, SA 5000 Australia; 4 Department of Otolaryngology, Head and Neck Surgery, Royal Adelaide Hospital, Adelaide, SA 5000 Australia; 5 Department of Surgery-Otolaryngology Head and Neck Surgery, The University of Adelaide, Adelaide, SA 5000 Australia

**Keywords:** Microbiome, 16S ribosomal RNA, Long read sequencing, Head and neck cancer

## Abstract

Describing the microbial community within the tumour has been a key aspect in understanding the pathophysiology of the tumour microenvironment. In head and neck cancer (HNC), most studies on tissue samples have only performed 16S rRNA short-read sequencing (SRS) on V3-V5 region. SRS is mostly limited to genus level identification. In this study, we compared full-length 16S rRNA long-read sequencing (FL-ONT) from Oxford Nanopore Technology (ONT) to V3-V4 Illumina SRS (V3V4-Illumina) in 26 HNC tumour tissues. Further validation was also performed using culture-based methods in 16 bacterial isolates obtained from 4 patients using MALDI-TOF MS. We observed similar alpha diversity indexes between FL-ONT and V3V4-Illumina. However, beta-diversity was significantly different between techniques (PERMANOVA - R^2^ = 0.131, *p* < 0.0001). At higher taxonomic levels (Phylum to Family), all metrics were more similar among sequencing techniques, while lower taxonomy displayed more discrepancies. At higher taxonomic levels, correlation in relative abundance from FL-ONT and V3V4-Illumina were higher, while this correlation decreased at lower levels. Finally, FL-ONT was able to identify more isolates at the species level that were identified using MALDI-TOF MS (75% vs. 18.8%). FL-ONT was able to identify lower taxonomic levels at a better resolution as compared to V3V4-Illumina 16S rRNA sequencing.

**Supplementary Information:**

The online version contains supplementary material available at 10.1007/s00203-024-03985-7.

## Introduction

The effect of tumour associated microbial communities on tumour biology is under intense investigation (Helmink et al. 2019; Cullin et al. 2021; Sepich-Poore et al. 2021; Yang et al. 2023a). To date, the tumour microbiome has been implicated in modulating anti-tumoural immune responses, chemotherapy efficacy, and tumour progression (Helmink et al. 2019; Cullin et al. 2021; Sepich-Poore et al. 2021). Apart from tissues, microbial signatures from other collection sites such as stool and saliva may have diagnostic or prognostic roles in various cancers (Thomas et al. 2019; Ratiner et al. 2023; Yang et al. 2023a). Together these studies demonstrate the potential impact of understanding the tumour microbiome in cancers. However, as a prerequisite to further research, it is critical to use the right tools for a robust microbiome identification.

DNA sequencing techniques such as targeted sequencing of the 16S ribosomal RNA (rRNA) gene, metagenomics, and to a lesser extent, meta-transcriptomics have been instrumental in microbiome identification (Cullin et al. 2021). Of these, Illumina based short-read sequencing (SRS) of the 16S rRNA has been widely adopted due to its relatively low cost and high throughput (Cullin et al. 2021; Kim et al. 2024). The 16S rRNA gene is approximately 1,500 to 1,600 base pairs (bp) long in most bacteria, and is composed of nine variable regions which allows taxonomical identification of microbial communities. Although sequencing all nine variable regions offers better taxonomic resolution, most studies usually sequence only a selection of variable regions, limiting the capacity of species level identification (Yeo et al. 2024).

In head and neck cancer (HNC), most studies on microbiome identification relied on SRS of the 16S V3-V5 regions (V3-V4: ~465 bp, V4: ~250 bp, V4-V5: ~392 bp) on tissues, swabs, saliva, and oral rinse (Ting et al. 2023; Yeo et al. 2024). Our recent meta-analysis of V3-V5 short-read Illumina sequencing datasets identified key oral microbes localised in HNC tumours (Yeo et al. 2024). However, taxonomic classifications were limited to the genus level, with species-specific contributions to HNC pathophysiology largely unknown (Curry et al. 2022; Yeo et al. 2024). Given that several oral species such as *Fusobacterium nucleatum* and *Porphyromonas gingivalis* can promote tumour progression and alter anti-tumour immunity (Lan et al. 2023), utilising cutting-edge technologies that can provide species level information will provide critical insights to the role of microbiome in HNC.

Long read sequencing (LRS) technologies from Oxford Nanopore Technologies (ONT) or Pacific Biosciences (PacBio) have been rapidly improving (ONT: Quality Score (Q-score) > 20, PacBio: Q-score > 33) and applied in the mainstream for various DNA sequencing applications, enabling sequencing of longer reads (> 10,000 bp) (Oehler et al. 2023). Importantly, its application in full-length 16S rRNA gene sequencing enables in-depth taxonomic classification (Oehler et al. 2023). Numerous studies have compared ONT LRS to Illumina based 16S rRNA gene sequencing in mock communities, swabs, and faecal samples (Shin et al. 2016; Acharya et al. 2019; Winand et al. 2019; Fujiyoshi et al. 2020; Heikema et al. 2020; Wei et al. 2020; de Siqueira et al. 2021; Low et al. 2021; Matsuo et al. 2021; Oberle et al. 2021; Park et al. 2021; Rozas et al. 2021; Szoboszlay et al. 2023; Connell et al. 2024). Four studies investigated the difference in beta-diversity (Heikema et al. 2020; de Siqueira et al. 2021; Szoboszlay et al. 2023; Connell et al. 2024), with two studies showing differences in beta-diversities between ONT and Illumina (Heikema et al. 2020; Szoboszlay et al. 2023). Two other studies measured sum of agreement at genera level (sum of the percentage of matching genera) and showed that the median microbiome agreement between ONT and Illumina groups ranged from 65 to 70% (Heikema et al. 2020; Connell et al. 2024). Moreover, many studies have analysed the differences or correlations between the abundance estimates generated by ONT and Illumina sequencing technologies at different taxonomic levels (Shin et al. 2016; Acharya et al. 2019; Winand et al. 2019; Fujiyoshi et al. 2020; Heikema et al. 2020; Wei et al. 2020; de Siqueira et al. 2021; Low et al. 2021; Matsuo et al. 2021; Oberle et al. 2021; Park et al. 2021; Rozas et al. 2021; Szoboszlay et al. 2023; Connell et al. 2024). The consensus is that at higher taxonomic levels, there were greater correlation observed, while the least correlation was observed at species level (Shin et al. 2016; Wei et al. 2020; Matsuo et al. 2021; Connell et al. 2024). To date, there has been no comparison between LRS and SRS using tumour tissue samples.

In this study, we comprehensively evaluated the differences in microbiome diversities and abundance between ONT and Illumina 16S rRNA sequencing technique on HNC tissue samples. Bacterial abundance between ONT and Illumina was evaluated at each taxonomic level using paired Wilcoxon test on relative abundance and paired ANOVA-Like Differential Expression tool 2 (ALDEx2) differential abundance analysis, which takes into account the compositional and zero-inflation nature of microbiome dataset (Fernandes et al. 2014). Furthermore, matrix assisted laser desorption ionization-time of flight mass spectrometry (MALDI-TOF MS) was also performed on bacteria isolated from 4 patient tissue samples for comparison to the 16S rRNA sequencing performed. To our best knowledge, this is the first study to perform long read 16S rRNA sequencing on HNC cancer tissue samples, and the first to evaluate ONT and Illumina 16S rRNA sequencing on HNC tissue samples.

## Methods

### Sample collections

Tumour samples were collected from 26 HNC patients undergoing surgical resection of primary tumours at the Royal Adelaide Hospital (Adelaide, SA, Australia) and The Memorial Hospital (Adelaide, SA, Australia). Tumour samples were placed into a sterile cryotube immediately after surgical excision to prevent any environmental contamination. Ethics approval for the collection and storage of patient samples were granted by Central Adelaide Local Health Network Human Research Ethics Committee (Adelaide, South Australia) (HREC MYIP14116), and all patients had signed written informed consent.

### DNA extraction

DNA was extracted in a laminar flow cabinet with aseptic technique, using DNeasy Blood & Tissue Kit (Qiagen, Germany, Hilden) with some modification, as described previously (Hang et al. 2014). Briefly, prior to DNA extraction, the tissue samples were homogenised using 3 mm stainless steel beads (Qiagen) and a TissueLyser II (Qiagen) at 23 Hz for 3 min. Afterwards, the homogenized tissues were incubated with 1 mg/mL lysozyme (cat no: L3790, Sigma Aldrich, MO, USA) and 0.2 mg/mL lysostaphin (L7386, Sigma) at 37 °C for 1 h, followed by 0.5 mg/mL proteinase K (Qiagen) incubation at 56 °C for 2 h, before proceeding with manufacturer’s DNA extraction protocol. The DNA was quantified using Qubit™ dsDNA Quantification Assay Kit (Invitrogen, USA, MA), before undergoing Illumina 16S rRNA V3-V4 SRS (referred to as V3V4-Illumina) and ONT full-length V1-V9 16S rRNA LRS (referred to as FL-ONT). Negative controls were also included in extraction process.

### V3V4-Illumina 16S rRNA sequencing

PCR amplification and sequencing was performed by the Australian Genome Research Facility (Adelaide, SA, Australia). PCR amplicons were generated using V3-V4 primers (341 F-CCTAYGGGRBGCASCAG, 806R-GGACTACNNGGGTATCTAAT) and conditions as described previously (Connell et al. 2024). Quality score and read length of raw reads were produced using qckitfastq and fastqc (Table S2).

### FL-ONT 16S rRNA sequencing

Full-length V1-V9 sequencing (27 F-AGAGTTTGATCMTGGCTCAG, 1492R -CGGTTACCTTGTTACGACTT) was performed using ONT MinION workflows (Oxford Nanopore Technologies, Oxford, UK). Full length 16S rRNA were amplified using 16S Barcoding Kit (SQK-16S024, Oxford Nanopore Technologies). Amplicons were purified using AMPure® XP beads (Beckman Coulter Diagnostics, USA, CA), quantified using Qubit HS kit (Qiagen), before sequencing on R9.4.1 chemistry (FLO-MIN106) flow cells (Oxford Nanopore Technologies), following manufacturer’s protocol. Basecalling was conducted using the super-accuracy basecalling model with Guppy v6.2.11. Quality score and read length of raw reads were produced using Nanoplot (Table S2) (De Coster and Rademakers 2023).

### Pre-processing and taxonomy assignment

For FL-ONT, filtlong was used to remove short and low quality reads from LRS (parameters: min_length = 1300, max_length = 1700, min_mean_q = 9). After filtering, EMU, was used to estimate full-length 16S rRNA relative abundance for FL-ONT (Curry et al. 2022). EMU was designed for full-length 16S rRNA with high error rate. It applies a two-stage process: (1) Performing proper alignments between reads and reference database, (2) Expectation–maximization algorithm based error-correction to refine species level relative abundance based on total read mapping counts (Curry et al. 2022). For V3V4-Illumina, primers were trimmed using QIIME2-cutadapt plugin (Martin 2011; Bolyen et al. 2019). Taxonomy assignment was performed using QIIME2 plugin, Divisive Amplicon Denoising Algorithm 2 (DADA2), as per author’s recommendation (maxEE = c(2,5) (Table S3) (Callahan et al. 2016; Bolyen et al. 2019). All taxonomic assignment were performed using SILVA reference database v138 (Quast et al. 2012). Paired samples with low read counts (< 1000) after taxonomy alignment were removed. Negative controls were filtered at this step, as they had no read counts.

### Alpha- and Beta-diversity analysis

Since short-read Illumina 16S rRNA sequencing is limited to genus level resolution (Curry et al. 2022), we performed alpha and beta-diversities analyses at the genus level. Before diversities analysis, samples were rarefied using rarefy_even_depth in phyloseq to sample with least depth (read = 1612) (McMurdie and Holmes 2013). Alpha-diversity was measured using Shannon, Simpson, InvSimpson and Observed indexes, using microeco R package (Liu et al. 2021). Wilcoxon matched-pairs signed rank test was performed to determine differences between paired samples sequenced using different techniques.

For beta-diversity analysis, rarefied relative abundance of all genera were ordinated using Bray-Curtis distance and plotted plotted on a principal coordinate analysis (PCoA) using phyloseq v1.46 and ggpubr v0.6 R packages (McMurdie and Holmes 2013). Permutational multivariate analysis of variance (PERMANOVA) and Analysis of similarities (ANOSIM), strata for paired sample, were performed to assess differences between in beta-diversity between paired ONT and Illumina sequencing groups (Dixon 2003). Additionally, we also included the W_d_ test, a test which is robust for heteroscedastic datasets, to determine differences in beta-diversity between ONT and Illumina (Hamidi et al. 2019). Variance between groups were measured using the betadisper test from vegan v2.6 (Dixon 2003). Permutations for all tests were set to *n* = 9999. Additional compositional approach was also performed for beta-diversity by performing central-log ratio (CLR) normalisation (offset = 0.5) of all counts at the genera level (Gloor et al. 2017). CLR abundance was coordinated using Euclidean distance and plotted on a principal coordinate analysis (PCoA) using phyloseq v1.46 and ggpubr v0.6 R packages (McMurdie and Holmes 2013) (Figure S1).

### Differential abundance analysis

We analysed differential abundance at all taxonomic levels – phylum, class, order, family, genus, and species. Data was agglomerated to specific levels before downstream analysis. For differential relative abundance analysis, data was normalized into relative abundance (%), and Wilcoxon matched-pairs signed rank test adjusted for Benjamini-Hochberg corrected false discovery rate (FDR) was used to determine differences between paired samples. Additionally, we also applied ALDEx2 differential abundance analysis, which uses a Monte Carlo Dirichlet sampling approach which considers the compositional and zero-inflation nature of microbiome dataset while to determining differences between ONT and Illumina sequencing group (Fernandes et al. 2014).

### Culture-based identification

Additional homogenised tumour tissues from four patients (PT09, PT14, PT15, PT17) were also cultured on sheep blood agar plates (Thermofisher) in an anaerobic hood at 37^o^C condition, 0% O_2_, 5% CO_2_, 2.5% H_2_. Bacteria isolates were sent for MALDI-TOF MS for identification.

## Results

### Patient demographics and sequencing

Tumour tissue samples from 26 HNC patients were collected for FL-ONT and V3V4-Illumina (Table S1). Nanopore generated an average total of 96,506 raw reads with a mean Q score per read of 12.3 (Table S2). The average length for Nanopore sequencing was 1276 nt. After filtering, Nanopore contained an average of 41,482 reads (average length = 1576 nt) with mean Q score per read of 12.6 (Table S2B). For Illumina sequencing, average total of 33,582 raw reads (Mean q-score per read: Forward = 31.2, Reverse = 28.7), with a consistent length of 300 nt (Table S2). Data were processed using DADA2 for Illumina or EMU for ONT data. The SILVA v138 rRNA database was used for taxonomy alignment for 16s rRNA data generated from both sequencing techniques. After sample filtering and processing and agglomerating to each taxonomic level, the total phyla, classes, orders, families, genera, and species detected were as follows: 10 phyla, 15 classes, 35 orders, 59 families, 92 genera, and 167 species. Among these, 1 phyla, 2 classes, 6 orders, 11 families, 21 genera, and 70 species were unique to FL-ONT, while 1 phyla, 1 classes, 5 orders, 10 families, 13 genera, and 42 species were unique to V3V4-Illumina sequencing (Supplementary Table S4–S9). The number of reads that at each taxonomic level were as follows: Phylum (FL-ONT: 1,109,264, V3V4-Illumina: 437,740), class (FL-ONT: 1,109,264, V3V4-Illumina: 437,740), order (FL-ONT: 1,109,264, V3V4-Illumina: 437,740), family (FL-ONT: 1,105,666, V3V4-Illumina: 437,740), genus (FL-ONT: 1,096,578, V3V4-Illumina: 431,448), and species (FL-ONT: 780,352, V3V4-Illumina: 145,238).

### FL-ONT and V3V4-Illumina 16S rRNA sequencing groups display comparable alpha diversity indexes at the genus level

**Fig. 1:**
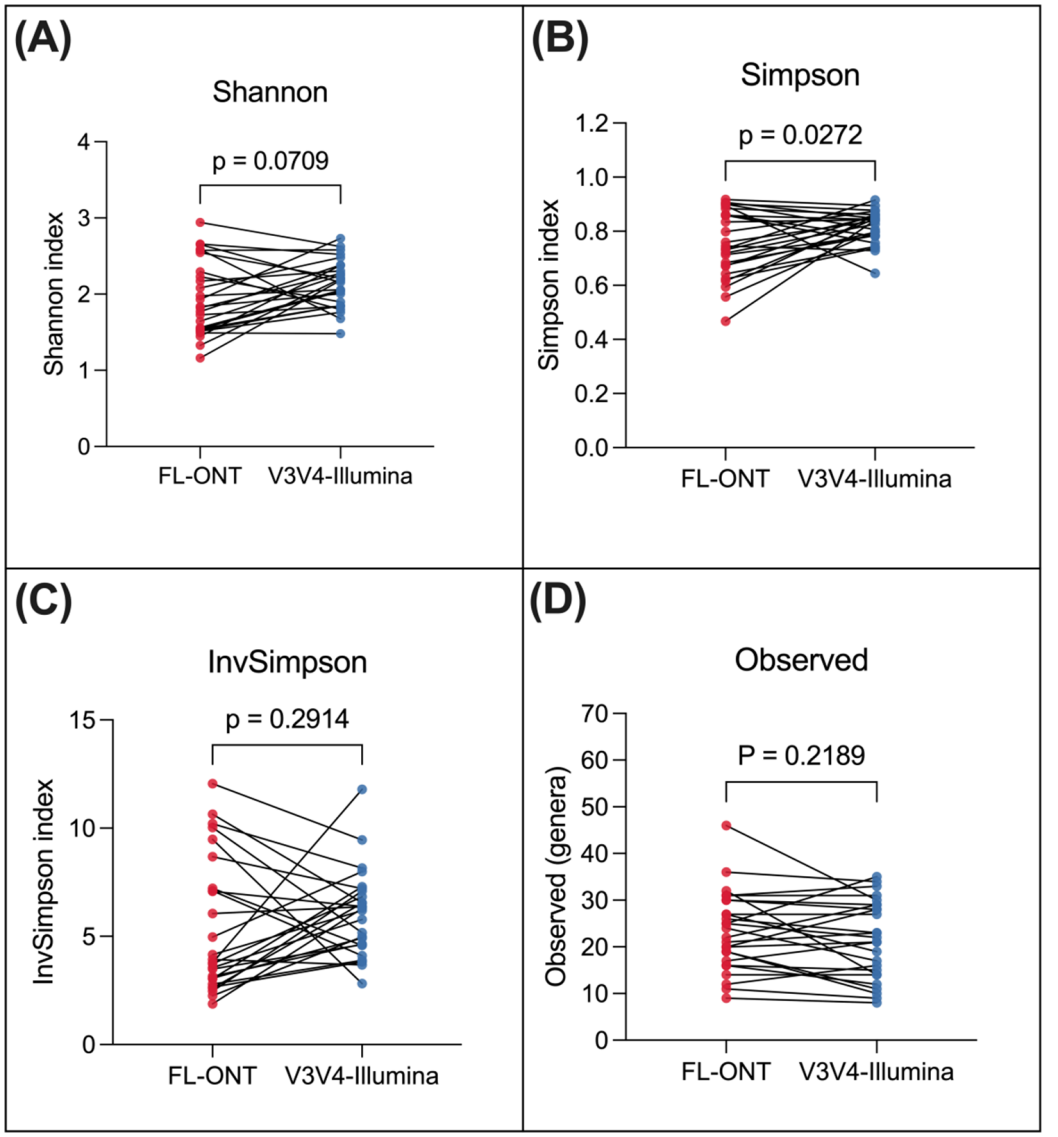
Paired alpha diversity analysis of FL-ONT and V3V4-Illumina at the genus level. Tissues were sequenced using ONT and Illumina technologies and data were aligned to the SILVA 16S rRNA database. To compare the differences in alpha diversity between technologies, paired Wilcoxon rank sum tests (adjusted for FDR) was performed for (**A**) Shannon index, (**B**) Simpson index, (**C**) InvSimpson, and (**D**) Observed genera using R package, microeco

To compare observed richness and evenness between FL-ONT and V3V4-Illumina, alpha diversity was measured using Shannon, Simpson, InvSimpson, and Observed indexes (Fig. 1). Since Illumina SRS 16S rRNA sequencing is largely limited to genus level resolution, alpha diversity was measured at genus level (Curry et al. 2022). After agglomerating rarefied datasets to genus level, a total of 92 genera were identified. Similar to previous findings comparing LRS and SRS (Heikema et al. 2020), we found no significant differences (*p* > 0.05) between ONT and Illumina 16S rRNA sequencing – Shannon (median difference = -0.207), InvSimpson (median difference = -1.140) and Observed genera (median difference = 1.00) (Fig. 1A, C and D). However, Simpson index (median difference = -0.078, *p* = 0.027) showed statistically significant but subtle differences between groups (Fig. 1B). Overall, these results suggest that there are subtle differences between ONT and Illumina 16S rRNA sequencing groups with respect to alpha diversity at the genus level.

### Differences in beta-diversity were observed between paired FL-ONT and V3V4-Illumina sequencing on tumour samples at the genus level

**Fig. 2:**
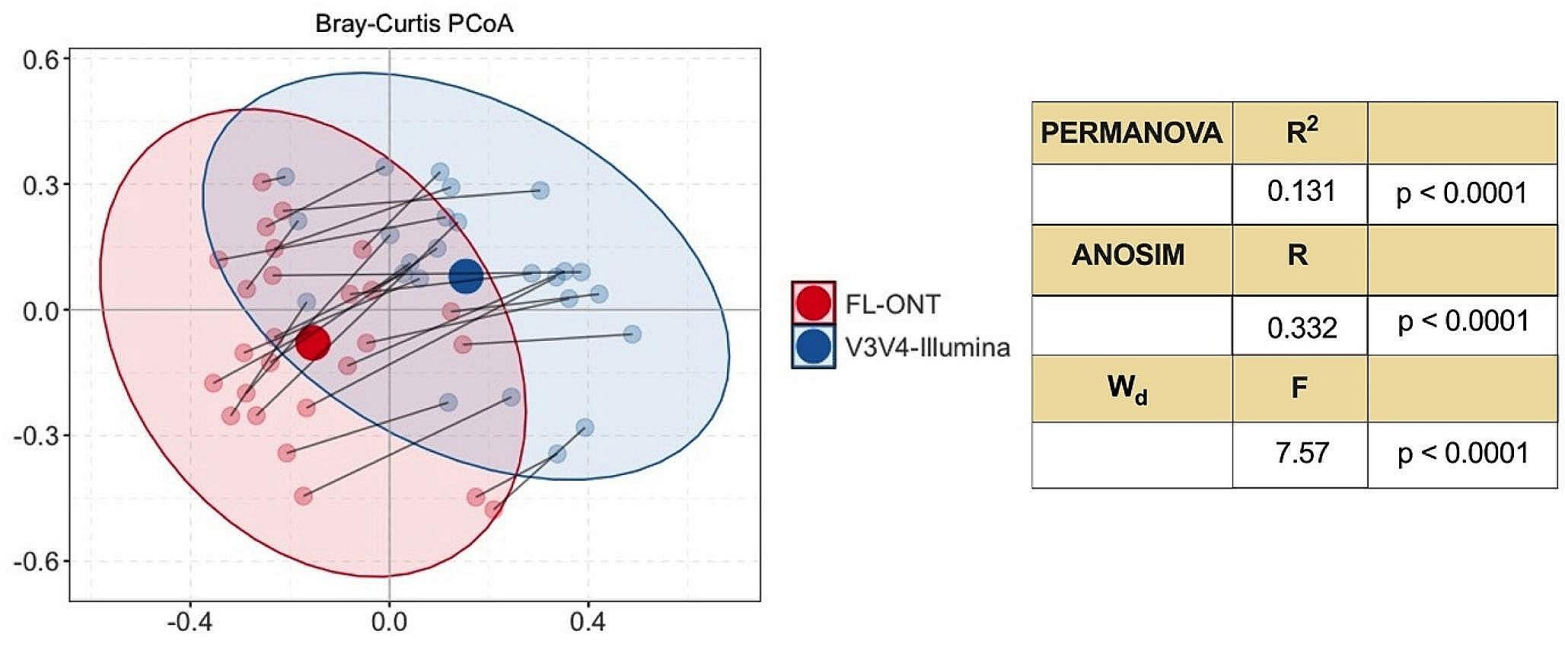
Paired beta diversity analysis of paired FL-ONT and V3V4-Illumina 16S rRNA sequencing on tissue samples at the genus level. Principal Coordinate Analysis (PCoA) plot of Bray-Curtis distance on rarefied normalized abundance. PERMANOVA, ANOSIM and W_d_ test were performed, statistics and p-value were presented. Red and blue dot-points represents ONT and Illumina 16S rRNA sequencing respectively, while line between dot-points represents paired samples

Differences in β-diversity between FL-ONT and V3V4-Illumina were assessed using PCoA plot of Bray-Curtis distance on rarefied relative abundance, PERMANOVA, ANOSIM and W_d_ test (Fig. 2). Ordination PCoA Bray-Curtis plot suggest that there is a shift in beta diversity between FL-ONT and V3V4-Illumina 16S rRNA sequencing (Fig. 2). Similarly, we observed significant differences in β-diversity between FL-ONT and V3V4-Illumina using PERMANOVA test (PERMANOVA – R^2^ = 0.131, F = 8.49, *p* < 0.0001). Dissimilarities between groups were assessed using an ANOSIM test (*R* = 0.332, *p* < 0.0001), further showing significant differences between both sample groups (Fig. 2). Furthermore, no significant differences in dispersion were observed between both technologies (Permutest – *p* = 0.854) (Table S10B). Further test using W_d_ test (a test robust for heteroscedastic datasets), we also showed significant differences between FL-ONT and V3V4-Illumina group (W_d_: F = 7.57, *p* < 0.0001) (Hamidi et al. 2019) (Fig. 2). Additionally, we also performed beta-diversity test using compositional approach (Figure S1, Table S10). Differences in β-diversity between FL-ONT and V3V4-Illumina were assessed using PcoA plot of Euclidean distance on CLR abundance (Figure S1, Table S10). Overall, we also observed similar findings with this approach (Figure S1, Table S10). Taken together, these findings show that β-diversity differs between FL-ONT and V3V4-Illumina 16S rRNA sequencing at the genus level.

### Paired sample analysis of FL-ONT and V3V4-Illumina 16S rRNA sequencing reveals decreasing correlation in relative abundance from higher to lower taxonomic levels

**Fig. 3:**
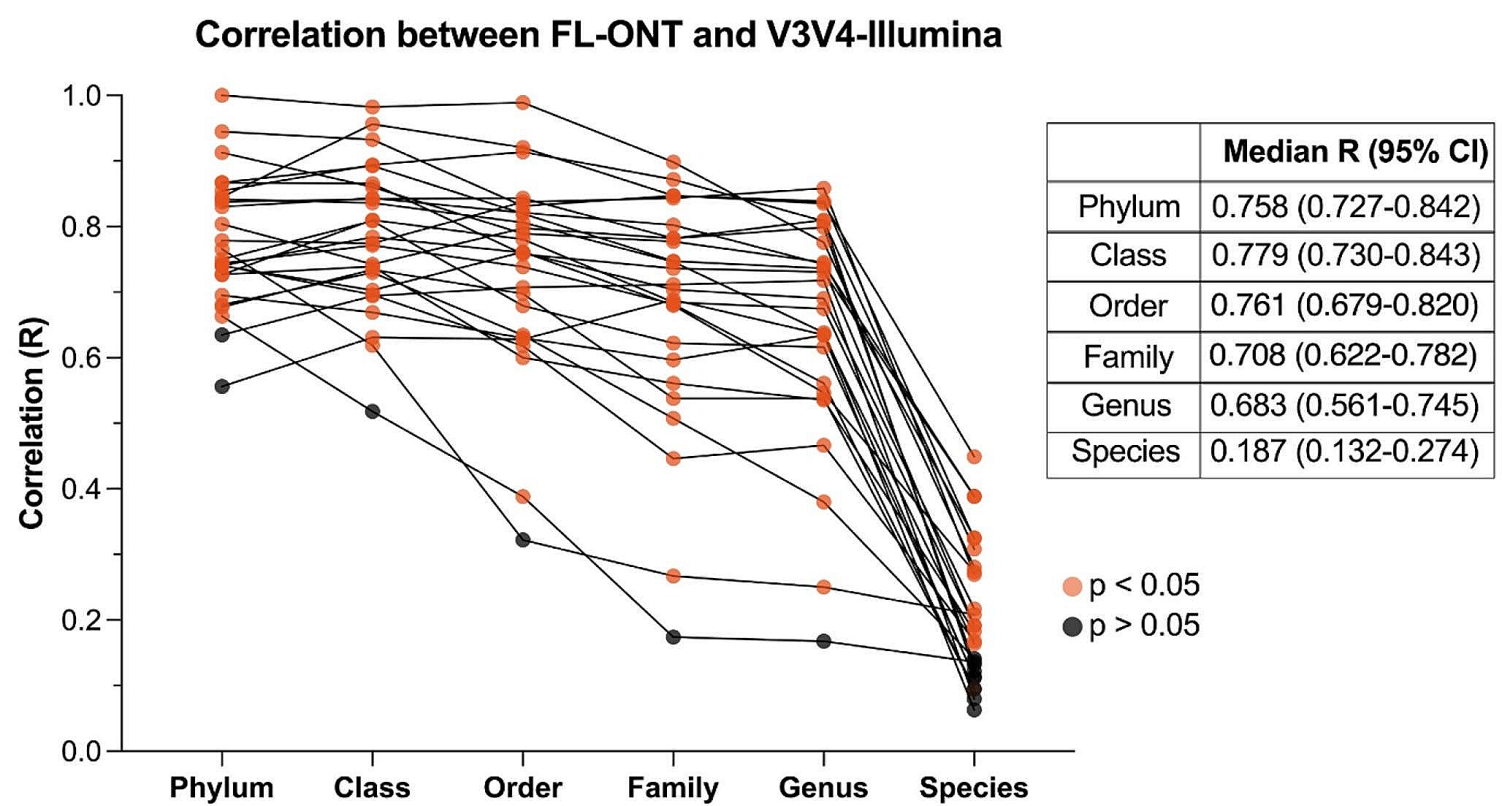
Correlation in bacterial relative abundance (%) at every taxonomic level between FL-ONT and V3V4-Illumina 16S rRNA sequencing. Spearman correlation analysis was performed for paired FL-ONT and V3V4-Illumina groups at each taxonomy level. Each point represents correlation value (R) between FL-ONT and V3V4-Illumina in each patient, and orange points represent *p* < 0.05

To determine taxonomic differences at each taxonomic level between FL-ONT and V3V4-Illumina sequencing technologies, we performed correlation analysis of relative abundance between paired samples, and paired Wilcoxon rank sum test on CLR-normalized abundance (ALDEx2) and on relative abundance (Fig. 3, Supplementary Table S4–S9, Supplementary Figure S2–S5) (Fernandes et al. 2014). Full description of taxonomic differences for phylum, class, order, and family level are presented in Supplementary Materials (Figure S2–S5). Overall, the bacteria identified by FL-ONT and V3V4-Illumina group were mostly from the same lineage at the phylum, class, order, and family taxonomical levels. However, we also detected bacteria that were unique to the sequencing technique, albeit detected at very low abundance (< 0.1%) (Table S4–S7). Furthermore, we also observed that there was a good concordance in the relative abundance of the top bacteria detected, whereby both techniques have similar top bacteria detected (Figure S2–S5).

We further performed correlation analysis of relative abundance between paired FL-ONT and V3V4-Illumina sequencing (Fig. 3). Moderate correlation in relative abundance(*R* > 0.7) were observed from phylum to family level, with phylum (*R* = 0.758), class (*R* = 0.779) and order (*R* = 0.761) level showing similar median R value as compared to family (*R* = 0.708) level (Fig. 3). Correlation in relative abundance continues to decrease from genus (*R* = 0.683) to species level. Notably, species level relative abundance showed significantly poorer correlation (*R* = 0.187, 95% CI = 0.132–0.274) compared to other taxonomic levels (Fig. 3).

### FL-ONT and V3V4-Illumina 16S rRNA sequencing displays greater discrepancies in microbial community profiling at the genus level

**Fig. 4:**
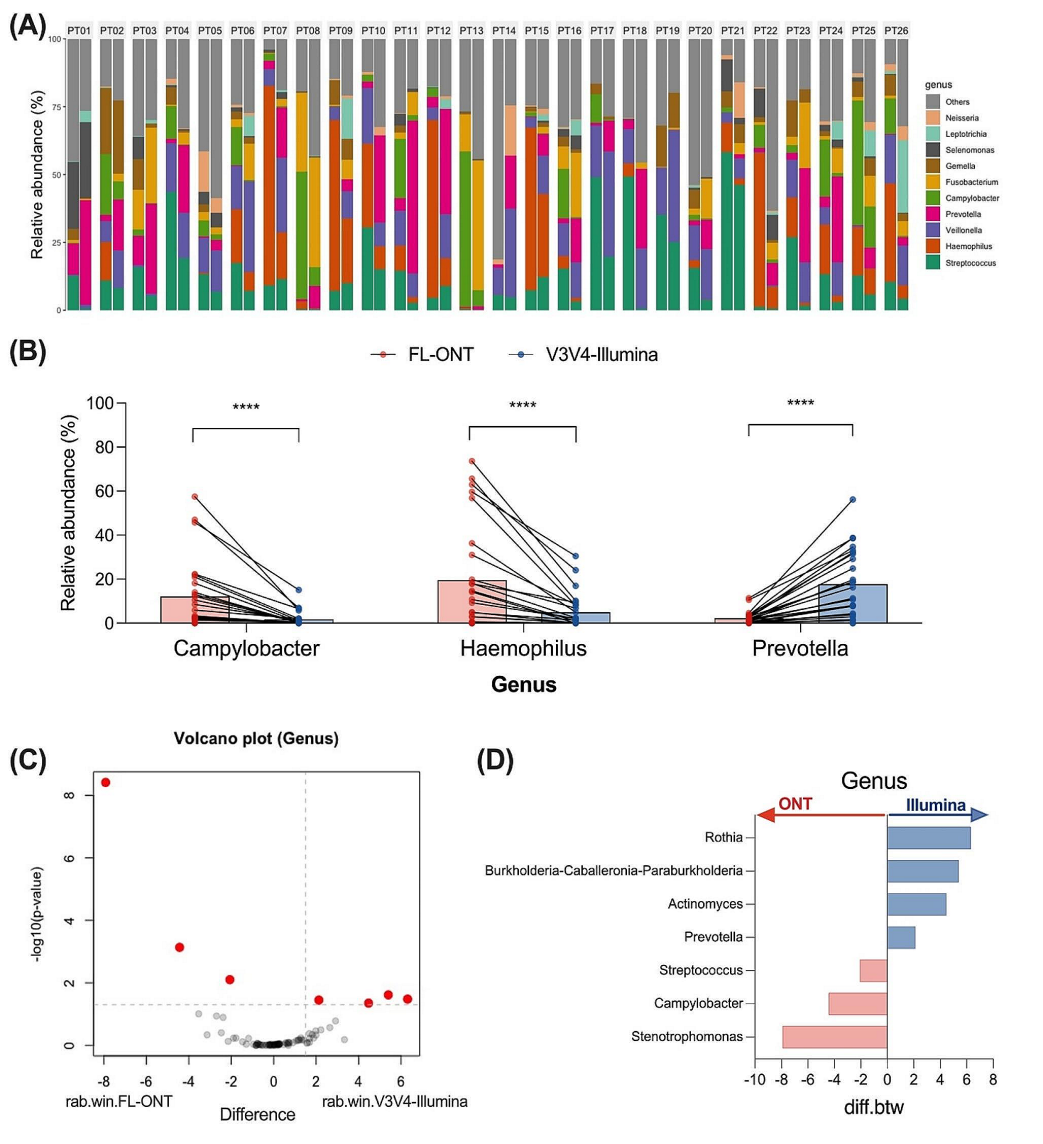
Comparison of abundance between FL-ONT and V3V4-Illumina 16S rRNA sequencing at the species level. After agglomerating to species level, a total of 167 species were identified. (**A**) Venn diagram of identified species in FL-ONT and V3V4-Illumina. Identified species is defined as having > 0% abundant (Table S9A). (**B**) Relative abundance (%) of significantly different species with mean differences > 10% between ONT and Illumina groups. (**C**) Relative abundance (%) of species that were detected in both techniques with mean differences > 1% between FL-ONT and V3V4-Illumina. Paired Wilcoxon test was performed to compare differences between FL-ONT to V3V4-Illumina sequencing. Additionally, ALDEx2 was performed to assess differences in species between sequencing techniques. (**D**) ALDEx2 volcano plot. Red dot points represent Benjamini-Hochberg corrected FDR p-value of Wilcoxon test < 0.05. Rab.win.group refers to median bacterial species clr value for the group of samples. **E**) Top 5 species detected based on effect size using ALDEx2 analysis. Diff.btw refers to median difference in bacterial species clr values between FL-ONT and V3V4-Illumina groups (Illumina - ONT). ****p < 0.0001, ***p < 0.001

**Fig. 5:**
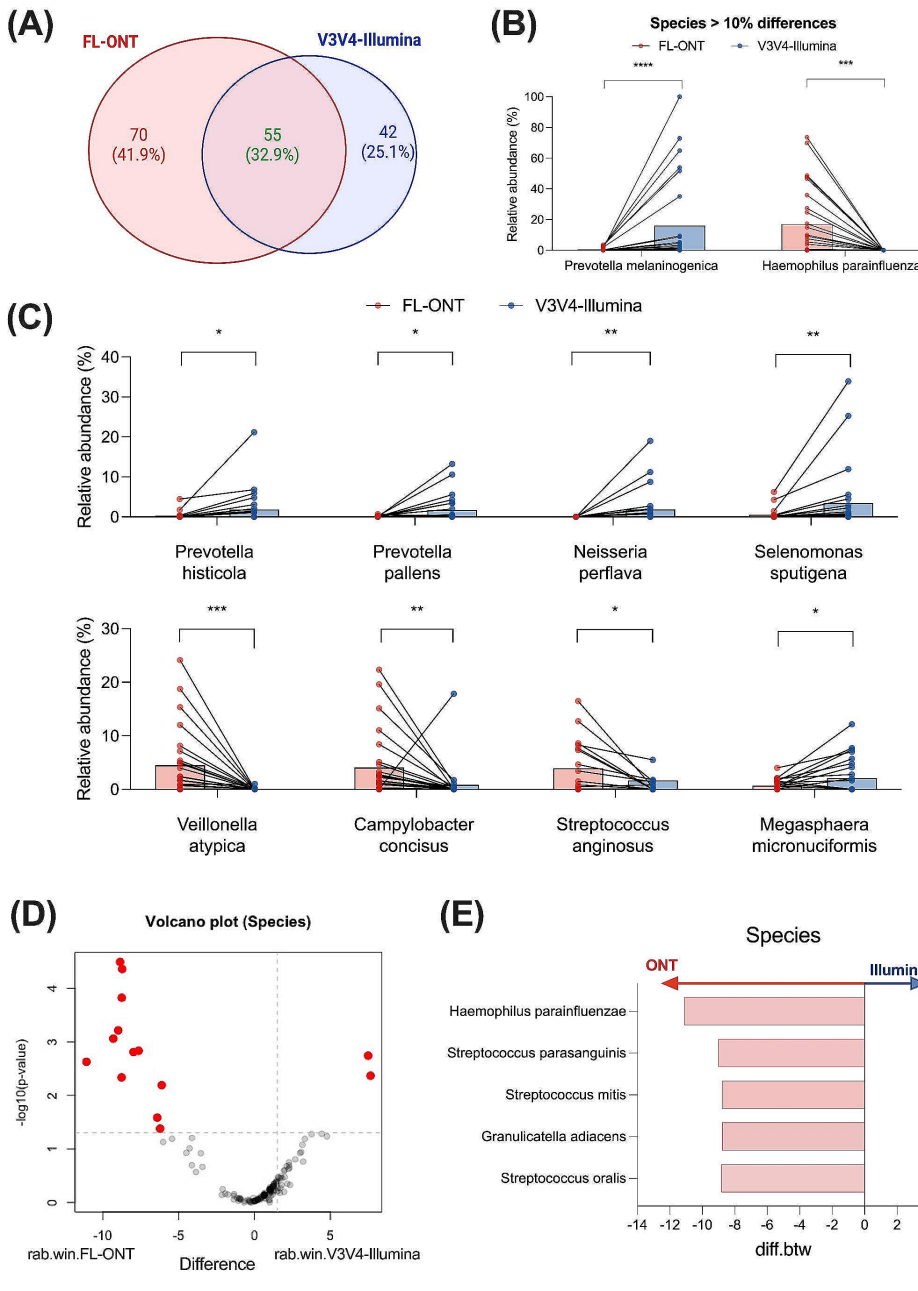
Comparison of abundance between FL-ONT and V3V4-Illumina 16S rRNA sequencing at the Genus level. After agglomerating to genus level, a total of 92 genera were identified. (**A**) Relative abundance (%) of top 10 genus, strata to per patient. For each patient panel, ONT and Illumina sequencing were represented by left and right bar plot respectively. (**B**) Relative abundance (%) of genus with > 10% differences between techniques. Paired Wilcoxon tests were performed to compare differences between ONT to Illumina sequencing. Additionally, ALDEx2 was performed to assess differences in genus between sequencing techniques. (**C**) ALDEx2 volcano plot. Red dot points represent Benjamini-Hochberg corrected FDR p-value of Wilcoxon test < 0.05. Rab.win.group refers to the median bacterial clr value for the group of samples. (**D**) Genera that were significantly different between ONT and Illumina using ALDEx2 analysis. Diff.btw refers to the median difference in bacterial clr values between ONT and Illumina groups (Illumina - ONT). ****p < 0.0001

Since Illumina 16S rRNA SRS is capable of identifying taxa mostly to the genus level, with limited capability of identification at species level, we compared FL-ONT and V3V4-Illumina at the genus level (Martínez-Porchas et al. 2016; Curry et al. 2022). When we compared relative abundance between sequencing techniques, we found that 29/92 bacterial genera were significantly different in relative abundance (Fig. 4A and B, Table S8A). *Haemophilus* (mean diff = 14.6%, *p* < 0.0001) and *Campylobacter* (mean diff = 10.5%, *p* < 0.0001) had significantly higher relative abundance in FL-ONT group, while *Prevotella* (mean diff = -15.4%, *p* < 0.0001) had significantly higher relative abundance in the V3V4-Illumina group (Fig. 5B, Table S8A). Other notable bacterial genera such as *Streptococcus* (mean diff = 9.71%, *p* < 0.0001) and *Fusobacterium* (mean diff = -6.72%, *p* = 0.00002) also had significantly higher relative abundances in FL-ONT and V3V4-Illumina group respectively (Table S8A). Of these 29 bacterial genera, 22 bacterial genera had less than 5% differences in relative abundance between techniques, although being statistically significant (Table S8A). Notably, a moderate correlation (Median *R* = 0.683, 95% CI = 0.561–0.745) between FL-ONT and V3V4-Illumina was observed at the genus level (Table S8B, Fig. 3). Among the top 10 genera detected in FL-ONT, only 6/10 genera were among the top genera detected in V3V4-Illumina (Table S8A).

Using ALDEx2 differential abundance test, 7/92 bacterial genera were significantly different in CLR-abundance between the sequencing technologies (Fig. 5C and D, Table S8C). *Streptococcus* (CLR_diff.btw_ = -2.07, effect size = -1.31), *Campylobacter* (CLR_diff.btw_ = -4.43, effect size = -1.75), *Stenotrophomonas* (CLR_diff.btw_ = -7.92, effect size = -2.80) were lower in CLR-abundance in V3V4-Illumina samples, while *Prevotella* (CLR_diff.btw_ = 2.13, effect size = 1.20), *Actinomyces* (CLR_diff.btw_ = 4.47, effect size = 0.97), *Burkholderia-Caballeronia-Paraburkholderia* (CLR_diff.btw_ = 5.40, effect size = 0.95), and *Rothia* (CLR_diff.btw_ = 6.31, effect size = 1.02) were higher in V3V4-Illumina samples (Fig. 5C and D, Table S8C). Among these bacteria genera, *Rothia* and *Burkholderia-Caballeronia-Paraburkholderia* were the only genera not detected in FL-ONT (Table S8A). Notably, the family of these 7 genera were also significantly different when comparing FL-ONT to V3V4-Illumina 16S rRNA sequencing (Table S7C).

### ONT LRS full-length 16S rRNA sequencing is superior for species level bacterial identification

Illumina SRS is limited to sequencing short fragments which results in poor capacity to differentiate and identify highly similar species (Martínez-Porchas et al. 2016; Curry et al. 2022). By sequencing the full-length 16S rRNA gene, FL-ONT is able to provide bacterial community identification at the species level. We further compared FL-ONT to V3V4-Illumina in HNC tissues samples at the species level. Furthermore, we also isolated bacteria from 4 HNC patients and identified these bacteria using MALDI-TOF MS to confirm that FL-ONT were able to identify the correct bacterial species.

A total of 167 bacteria species were identified among both sequencing groups (Table S9A). Of these 167 bacterial species detected, 55/167 (32.9%) were identified by both sequencing approaches (Fig. 4A). We detected 70/167 (41.9%) bacterial species that were unique to the FL-ONT group, with 49/70 of these bacterial species containing less than 1% of abundance (Fig. 4A, Table S9A). For V3V4-Illumina sequencing, 42/167 (25.1%) species were unique to the group and critically all 35 of these bacterial species were less than 1% abundant in V3V4-Illumina group (Fig. 4A, Table S9A).

Based on relative abundance, we identified 50 bacterial species to be significantly different (*p* < 0.05) between sequencing groups (Table S9A). *Prevotella melaninogenica* and *Haemophilus parainfluenzae* were the only bacterial species that displayed more than 10% differential relative abundance between techniques (Fig. 4B). Of these 50 bacterial species, V3V4-Illumina were only able to detect 21 bacterial species, while 41 bacterial species were detected by FL-ONT (Table S9A). Bacterial species that were not detected by V3V4-Illumina includes notable species such as *Haemophilus parainfluenzae*, *Fusobacterium nucleatum*, and *Streptococcus parasanguinis*, which were detected at 17.1%, 3.8% and 3.3% respectively, within FL-ONT samples (Fig. 4B, Table S9A). When comparing bacterial species that were present in both techniques, *Prevotella melaninogenica* was significantly elevated (mean diff > 10%, *p* < 0.05) in V3V4-Illumina samples (mean diff = -15.4%, *p* < 0.0001) (Fig. 4B). Additionally, other species present in both techniques that were substantially differences (> 1%, *p* < 0.05) between FL-ONT and V3V4-Illumina groups includes *Prevotella histicola* (mean diff = -1.56%), *Prevotella pallens* (mean diff = -1.62%), *Neisseria perflava* (mean diff = -1.90%), *Selenomonas sputigena* (mean diff = -2.94%), *Veillonella atypia* (mean diff = 4.45%), *Campylobacter concisus* (mean diff = 3.24%), *Streptococcus anginosus* (mean diff = 2.25%), *and Megasphaera micronuciformis* (mean diff = -1.44%) (Fig. 4C, Table S9A). As expected, we observed a poor correlation between FL-ONT and V3V4-Illumina at the species level (*R* = 0.187, 95% CI = 0.132–0.274) (Fig. 3).

Using ALDEx2 differential abundance analysis, 16/167 species were significantly different among techniques (Fig. 4D, Table S9C). Based on effect size, the top 5 signifcantly different ALDEx2 species were *Streptococcus oralis* (CLR_diff.btw_ = -8.84, effect size = 3.90), *Granulicatella adiacens* (CLR_diff.btw_ = -8.77, effect size = -2.03), *Streptococcus mitis* (CLR_diff.btw_ = -8.80, effect size = -1.98), *Streptococcus parasanguinis* (CLR_diff.btw_ = -9.01, effect size = -1.78), *Haemophilus parainfluenzae* (CLR_diff.btw_ = -11.1, effect size = -1.64), all of which elevated in FL-ONT (Fig. 4D and E, Table S9C). Furthermore, MALDI-TOF MS identified 16 isolates from four patients (Table S11). Overall, 75% of total isolates identified by MALDI-TOF MS were also identified using FL-ONT, while V3V4-Illumina was able to only identify 18.8% of isolates (Table S11A).

As expected, we observed most discrepancies in bacterial species identification between FL-ONT and V3V4-Illumina sequencing groups. Moreover, FL-ONT was able to identify more unique bacterial species at a higher bacterial abundance than V3V4-Illumina sequencing. Furthermore, MALDI-TOF MS identification were more identical to FL-ONT than V3V4-Illumina. Similar to previous studies, poorest correlation between FL-ONT and V3V4-Illumina was observed at species level (Fig. 3) (Shin et al. 2016; Wei et al. 2020; Matsuo et al. 2021).

## Discussion

Recent studies suggest that microbiome contributions to tumour pathobiology can be attributed to specific bacterial species (Helmink et al. 2019; Cullin et al. 2021; Sepich-Poore et al. 2021), there is a significant need to adopt sequencing technologies capable of species level identification such as FL-ONT 16S rRNA sequencing (Curry et al. 2022). We have previously reported a consensus tissue microbiome signature in HNC using previously published Illumina SRS 16S rRNA sequencing data (Yeo et al. 2024). To the best of our knowledge, this is the first study to perform FL-ONT 16S rRNA sequencing on HNC tumour samples. Furthermore, we comprehensively assessed the performance of FL-ONT to V3V4-Illumina sequencing. We found that alpha diversity was comparable between paired FL-ONT and V3V4-Illumina 16S rRNA sequencing. In contrast, beta-diversity was significantly different between paired FL-ONT and V3V4-Illumina 16S rRNA sequenced HNC samples. At higher taxonomic levels (phylum, class, order, and family), moderate correlations between the two sequencing methodologies for bacterial relative abundance, while at lower taxonomic levels, particularly species level, the correlations were poor. Importantly, FL-ONT identified more unique species that were also detected at higher in abundance than V3V4-Illumina.

In this study, we compared alpha and beta-diversities between FL-ONT to V3V4-Illumina 16S rRNA sequencing data at the genus level which is the current acceptable limit for short-read Illumina 16S rRNA sequencing based taxonomic classification (Curry et al. 2022). Similar to our previous work on nasal swabs (Connell et al. 2024), we identified comparable alpha-diversities between paired FL-ONT and V3V4-Illumina 16S rRNA sequencing in HNC tissues samples. Out of the 4 alpha diversities matrices tested, only Simpson index showed a statistically significant, but minimal difference (mean differences = -0.07) in our study. A previous study also reported minimal but statistically significant differences in alpha-diversity measurement using InvSimpson index between the two sequencing techniques (Heikema et al. 2020). Importantly, in our HNC tissue samples, we showed minimal or no differences in alpha-diversities. Consistent with our findings, previous reports have shown significant beta-diversity differences between ONT and Illumina based 16S rRNA sequencing in the gut and nasal microbiome (Heikema et al. 2020; Szoboszlay et al. 2023). Critically, our beta-diversities were stratified for patients accounting for inter-patient sample differences. Together, these findings indicate that ONT and Illumina 16S rRNA sequencing have minimal impact on bacterial genera richness and evenness, however overall bacterial composition was affected by the sequencing technique employed.

We next determined whether bacterial composition difference observed were present in every taxonomic level. Previous studies have examined FL-ONT and Illumina 16S rRNA sequencing datasets for differences in relative abundance at the phylum (Szoboszlay et al. 2023), order (Shin et al. 2016), family (Shin et al. 2016; Acharya et al. 2019; Winand et al. 2019; Connell et al. 2024), genus (Shin et al. 2016; Acharya et al. 2019; Winand et al. 2019; Fujiyoshi et al. 2020; Heikema et al. 2020; Wei et al. 2020; de Siqueira et al. 2021; Low et al. 2021; Matsuo et al. 2021; Oberle et al. 2021; Rozas et al. 2021; Connell et al. 2024), and species (Shin et al. 2016; Winand et al. 2019; Wei et al. 2020; Low et al. 2021; Connell et al. 2024) level. However, these studies have used different analytical approaches that may affect the interpretation of their results. Some compared relative abundance of paired samples without paired differential abundance analysis (de Siqueira et al. 2021; Oberle et al. 2021; Szoboszlay et al. 2023), while others compared averages within each sequencing group (Heikema et al. 2020; Wei et al. 2020). Most compared correlation in abundance between ONT and Illumina (Shin et al. 2016; Wei et al. 2020; Matsuo et al. 2021; Rozas et al. 2021), specifically the top 10 to 15 bacteria (Shin et al. 2016; Acharya et al. 2019; Wei et al. 2020; Matsuo et al. 2021), thus not reflecting the magnitude of differences in abundance between sequencing techniques. Furthermore, a few studies had small sample sizes (< 10) which limits their interpretation (Shin et al. 2016; Fujiyoshi et al. 2020; Oberle et al. 2021; Szoboszlay et al. 2023). Notably, in addition to this study, our previous study on nasal swabs was the only study to have applied paired analysis to evaluate differences in relative abundance (family, genus) and diversities between ONT and Illumina sequencing (Connell et al. 2024). Paired differential abundance analysis should be employed to account for inter-sample differences such as lifestyle activities including smoking, alcohol or diet intake that is known to affect the microbiome (Yu et al. 2017; Fan et al. 2018; Shoer et al. 2023).

Consistent with most studies (Shin et al. 2016; Wei et al. 2020; Matsuo et al. 2021), we observed a decrease in correlation between relative abundance produced from different sequencing techniques from higher to lower taxonomic levels (Figure S1). In our study, we observed differences in relative abundance between FL-ONT and V3V4-Illumina 16S rRNA sequencing especially for bacteria related to phylum *Campylobacterota, Proteobacteria, Actinobacteriota and Firmicutes*. Furthermore, we found that there were biases in the bacteria detected in FL-ONT or V3V4-Illumina 16S rRNA sequencing. ALDEx2 is an alternative method that considers compositional and zero-inflated microbiome datasets and is more robust than standard relative abundance analyses (Nearing et al. 2022). Using ALDEx2, we also identified differences at every taxonomy although at a smaller number, reflective of its conservative nature to reduce false-postives detection (Gloor et al. 2017; Nearing et al. 2022). Taken together, we have comprehensively shown that there are significant differences in the two sequencing technologies’ ability to detect the bacteria relative abundance of HNC tissues.

The microbiome has been reported to influence numerous facets of tumour pathobiology biology including treatment efficacy, tumour immunity and tumour progression (Yang et al. 2023a). Gemcitabine, a chemotherapeutic treatment for pancreatic, bladder and metastatic triple-negative breast cancers, can be transported into the cytoplasm of *Gammaproteobacteria* (class) using nucleoside transporter (NupC), where it gets inactivated by bacterial cytidine deaminase (Geller et al. 2017; Gallagher et al. 2022; Yang et al. 2023b). Gut-derived *Bifidobacterium* spp. is associated with increased response rates and progression free survival to PD-1 checkpoint inhibitors (Dizman et al. 2022). Notably, well-studied microbial metabolites such as butyrate, can also improve PD-1 checkpoint inhibitor response rates (Gopalakrishnan et al. 2018; Zhu et al. 2023). Butyrate can be produced from *Faecalibacterium* (genus) and *Akkermansia muciniphila* (species) (Gopalakrishnan et al. 2018; Zhu et al. 2023). Of note, these tumour modulating abilities is dependent on specific genomic features shared within a taxonomic level (Yang et al. 2023b). Thus, microbiome identification at higher taxonomical levels that can be accurately identified by Illumina 16S rRNA sequencing is important (Kim et al. 2024). However, our study shows that FL-ONT 16S rRNA sequencing is similar to the precision of V3V4-Illumina at higher taxonimical levels but with the advantage of providing species level identification in a cost-effective manner. A major benefit for ONT sequencers is the low base-cost (portable MinION and MinION Mk1C sequencers: ~$1000-$5000USD, GridION: ~$50,000USD), compared to its other counterparts (Illumina Miseq: ~$100,000USD, PacBio Sequel II system: ~$500,000USD) (Connell et al. 2024). High base cost may force independent research lab to outsource their sequencing. Additionally, ONT also have one of the lowest cost per 10 K reads (ONT R10.4.1: ~$6.91USD, ONT R9.4.1:~$6.22USD, PacBio:~$27.65USD, Novaseq:~$4.15USD (Zhang et al. 2023). Using bacterial isolate cell culture and MALDI-TOF MS identification, we also showed that FL-ONT was able to identify more bacterial isolates compared to V3V4-Illumina sequencing. Thus, at higher taxonomic levels, Illumina 16S rRNA remains a cost-effective and accurate method to screen for microbial community (Tedersoo et al. 2021; Kim et al. 2024). However, with continued advances in the development of LRS technologies full-length 16S rRNA sequencing is quickly becoming a more attractive alternative with its ability for species level microbial community classification (Tedersoo et al. 2021; Kim et al. 2024).

Although we have thoroughly investigated differences in both techniques, there are limitations to this study. While biological replicates were included, this study lacks technical replicates for each sequencing patient’s sample which will provide more confidence in the study. In addition, this study did not include an oral mock microbial community as a reference. Having a commercial oral mock community will allow benchmarking of library preparation steps such as primer efficacy and PCR conditions between both FL-ONT and V3V4-Illumina 16S rRNA sequencing. Furthermore, future studies should consider including other primers or all primer sets to cover the entire region of the 16S rRNA for short-read Illumina sequencing. This will ensure better coverage and comparison between full-length ONT and full-length short-read Illumina sequencing (Johnson et al. 2019). Additionally, future studies should also include more samples and culture conditions (i.e. aerobic and anaerobic) in the MALDI-TOF culturomics approach to provide substantial confidence in sequencing results. Lastly, adding on a metagenomics approach can also provide greater confidence with extra sequencing coverage outside of the 16S rRNA gene (Kim et al. 2024). In the context of HNC pathobiology, future addition of matched non-cancer and cancer samples could provide more insights to microbial differences at the species level.

In conclusion, our study provides the first comprehensive comparison of FL-ONT and V3V4-Illumina 16S rRNA microbial sequencing in HNC tumour tissue samples. We have shown that there were key differences such as beta-diversity and some bacterial groups in every taxonomy at every level. Critically, we show that FL-ONT can provide more information about the microbiome that is cost-effective. We expect this technology to be more widely adopted in future cancer microbiome studies.

### Electronic supplementary material

Below is the link to the electronic supplementary material.

Supplementary Material 1

Supplementary Material 2

## Data Availability

All sequencing data in this paper have been uploaded in NCBI SRA under bioproject accession number accession PRJNA1087430, and release upon publication.
