# Supplementary material for: A comparison between full-length 16S rRNA Oxford nanopore sequencing and Illumina V3-V4 16S rRNA sequencing in head and neck cancer tissues
Source: Arch Microbiol. 2024 May 7;206(6):248. doi: 10.1007/s00203-024-03985-7 (PMC11076400; doi:10.1007/s00203-024-03985-7)
Supplement: Supplementary file 2 — Supplementary Material 2 [file 203_2024_3985_MOESM2_ESM.docx]

**Supplementary Materials:**

1. **Supplementary text:**
2. **Beta-diversity analysis using CLR normalized abundance.**

Differences in β-diversity between FL-ONT and V3V4-Illumina were assessed using PCoA plot of Euclidean distance on CLR normalized abundance, PERMANOVA, ANOSIM and Wd test (Figure S1). Ordination PCoA Euclidean plot suggest that there is a shift in beta diversity between FL-ONT and V3V4-Illumina 16S rRNA sequencing (Figure S1). Similarly, we observed significant differences in β-diversity between FL-ONT and V3V4-Illumina using PERMANOVA test (PERMANOVA - R^2^ = 0.084, p < 0.0001). Dissimilarities between groups were assessed using an ANOSIM test (R = 0.238, p < 0.0001), further showing significant differences between both sample groups (Figure S1). No significant differences in dispersion were observed between both technologies (Permutest – p > 0.19) (Table S10). We have additionally incorporated Wd test, a test which is robust for heteroscedastic datasets (Hamidi et al. 2019). Similar to PERMANOVA and ANOSIM, Wd test also showed significant differences between in β-diversity between FL-ONT and V3V4-Illumina 16S rRNA sequencing (Wd= 4.578, p = 0.0001) (Figure S1). Taken together, these findings show that β-diversity differs between FL-ONT and V3V4-Illumina 16S rRNA sequencing at the genus level.

1. **Paired sample analysis of FL-ONT and V3V4-Illumina 16S rRNA sequencing reveals differences at higher taxonomic levels - phylum, class, order, and family.**

To determine taxonomic differences at phylum, class, order, and family level between FL-ONT and V3V4-Illumina sequencing technologies, we performed paired Wilcoxon rank sum test on relative abundance and CLR-normalized abundance using ALDEx2 (Figure S2, Supplementary Table S4-S7, Supplementary Figure S2-S3) (Fernandes et al. 2014).

***Phylum level:***

Based on relative abundance, the most abundant phylum detected in both techniques were *Campylobacterota* (FL-ONT: 12.2%, V3V4-Illumina: 1.73%)*, Fusobacteriota* (FL-ONT: 3.51%, V3V4-Illumina: 13.8%)*, Bacteroidota* (FL-ONT: 4.13%, V3V4-Illumina: 25.9%)*, Proteobacteria* (FL-ONT: 28.9%, V3V4-Illumina: 11.5%)*, Firmicutes* (FL-ONT: 50.7%, V3V4-Illumina: 39.0%) (Figure S2, Table S4A). *Proteobacteria* (mean diff = 17.5%, p < 0.0001)*, Firmicutes* (mean diff = 11.7%, p < 0.01)*, and Campylobacterota* (mean diff = 10.4%, p < 0.0001) were significantly more abundant in FL-ONT group, while *Fusobacteriota* (mean diff = -10.3%, p < 0.0001) and *Bacteroidota* (mean diff = -21.7%, p < 0.0001) were more abundant in V3V4-Illumina group (Figure S2-3, Table S4A). Overall, FL-ONT and V3V4-Illumina sequencing showed a moderate correlation (R > 0.7) between groups at the phylum level (median R = 0.758, 95% CI= 0.727-0.842) (Figure 3, Table S4B). Furthermore, among top 5 phyla detected in FL-ONT, 4/5 phyla were also top phyla detected in V3V4-Illumina (Table S4A). Additionally, we applied ALDEx2 differential analysis and identified significantly (p < 0.05) lower CLR-abundance of *Campylobacterota* (CLR_diff.btw_ = -5.42, effect size = -2.36)*, Proteobacteria* (CLR_diff.btw_ = -3.85, effect size = -2.54)*, Firmicutes* (CLR_diff.btw_ = -2.38, effect size = -1.42)*,* and higher CLR-abundance in *Actinobacteriota* (CLR_diff.btw_ = 4.63, effect size = 1.33) in V3V4-Illumina, as compared to FL-ONT (Figure S4A, Table S4C).

***Class level:***

Based on relative abundance, we found that 12/15 bacterial classes were significantly different among sequencing groups, and 5 of these classes had a have mean difference of more than 10% (Figure S2-S3, Table S5A). The FL-ONT group had greater abundance of *Gammaproteobacteria* (mean diff = 17.2%, p < 0.0001)*, Bacilli* (mean diff = 13.6%, p < 0.0001)*, and* *Campylobacteria* (mean diff = 10.4%, p < 0.0001), while *Bacteroidia* (mean diff = -21.7%, p < 0.0001) and *Fusobacteriia* (mean diff = -10.3%, p < 0.0001) were greater in V3V4-Illumina group (Figure S2-S3, Table S5A). Overall, FL-ONT and V3V4-Illumina sequencing showed moderate correlation (R > 0.7) between groups at the class level (median R = 0.779, 95% CI= 0.730-0.843) (Figure 3, Table S5B). Moreover, among top 5 classes detected in FL-ONT, 3/5 classes were also among the top classes detected in V3V4-Illumina (Table S5A). Using ALDEx2, we found 6/15 bacterial classes that were significantly different (p < 0.05) between sequencing technologies (Figure S4, Table S5C). *Campylobacteria* (CLR_diff.btw_ = -5.28, effect size = -2.39)*, Gammaproteobacteria* (CLR_diff.btw_ = -3.63, effect size = -2.49), *Bacilli* (CLR_diff.btw_ = -3.03, effect size = -1.84)*, Clostridia* (CLR_diff.btw_ = -2.56, effect size = -1.55)*, Negativicutes* (CLR_diff.btw_ = -1.15, effect size = -0.676) were significantly lower CLR-abundance in V3V4-Illumina group, while V3V4-Illumina group were determined to contain more *Actinobacteria* (CLR_diff.btw_ = 5.18, effect size = 1.28) (Figure S4, Table S5C). Notably, all six bacterial classes were lineage to Phylum *Campylobacterota, Proteobacteria, Actinobacteriota* *and Firmicutes* (Table S4-S5). Similarly, there were bacterial classes that were only detected in FL-ONT or V3V4-Illumina groups, albeit being < 1% mean relative abundance (Table S5A).

***Order level:***

When comparing relative abundance at the order level, we identified 18/35 orders being significantly different between FL-ONT and V3V4-Illumina 16S rRNA sequencing group (Figure S2-S3, Table S6A). *Enterobacterales* (mean diff = 18.2%, p < 0.0001)*, Lactobacillales* (mean diff = 11.3%, p < 0.0001)*, Campylobacterales* (mean diff = 10.4%, p < 0.0001) were significantly higher in FL-ONT groups, while *Fusobacteriales* (mean diff = -10.3%, p < 0.0001) and *Bacteroidales* (mean diff = -19.3%, p < 0.0001) were higher in V3V4-Illumina sample group (Figure S2-S3, Table S6A). Overall, the correlation between FL-ONT and V3V4-Illumina at the order level (median R = 0.761, 95% CI= 0.679-0.820) similar to phylum and class levels (Table S6B, Figure 3). Among the top 10 bacteria order detected in FL-ONT, 9/10 were also top order detected in V3V4-Illumina (Table S6A). Using ALDEx2 differential abundance analysis, we identified 9/35 bacterial orders that were significantly different (p < 0.05) between sequencing technologies (Figure S4, Table S6C). *Xanthomonadales* (CLR_diff.btw_ = -8.07, effect size = -2.71)*, Campylobacterales* (CLR_diff.btw_ = -4.67, effect size = -2.11)*, Enterobacterales* (CLR_diff.btw_ = -3.64, effect size = -2.09)*, Lactobacillales* (CLR_diff.btw_ = -2.54, effect size = -1.57)*, Staphylococcales* (CLR_diff.btw_ = -2.53, effect size = -1.55)*,* and *Peptostreptococcales-Tissierellales* (CLR_diff.btw_ = -2.31, effect size = -0.99) have lower CLR-abundance in V3V4-Illumina samples, while *Micrococcales* (CLR_diff.btw_ = 6.17, effect size = 1.05), *Actinomycetales* (CLR_diff.btw_ = 4.35, effect size = 1.10), and *Bacteroidales* (CLR_diff.btw_ = 1.77, effect size = 1.42) were more abundant in V3V4-Illumina group (Figure S2-S3, Table S6C). All these orders were lineages of classes that were significantly different between sequencing technique groups (Table S5-S6). However, order *Micrococcales* were the only detected in V3V4-Illumina sequencing group.

***Family level:***

When comparing differences in relative abundance between FL-ONT and V3V4-Illumina groups, we identified differences in 29/59 families (Figure S2, Table S7A). Of these families, *Pasteurellaceae* (mean diff = 16.7%, p < 0.0001)*,* and *Campylobacteraceae* (mean diff = 10.4%, p < 0.0001) were significantly more abundant (> 10% differences, p < 0.05) in FL-ONT groups, while *Prevotellaceae* (mean diff = -17.0%, p < 0.0001) was significantly more abundant in V3V4-Illumina groups (Figure S3, Table S7A). Overall, the correlation between FL-ONT and V3V4-Illumina at the family level (median R = 0.708, 95% CI= 0.622-0.782) were lower than all the higher taxonomic levels (Figure 3, Table S7B). Among the top 10 bacteria families detected in FL-ONT, 7/10 were also top families detected with V3V4-Illumina (Table S7A). Using ALDEx2 differential abundance analysis, 11/59 bacterial families were significantly different (p < 0.05) between sequencing technologies. The V3V4-Illumina group exhibited lower CLR-abundance of *Xanthomonadaceae* (CLR_diff.btw_ = -8.11, effect size = -2.82)*, Anaerovoracaceae* (CLR_diff.btw_ = -4.61, effect size = -0.828)*, Campylobacteraceae* (CLR_diff.btw_ = -4.56, effect size = -1.97)*, Pasteurellaceae* (CLR_diff.btw_ = -3.85, effect size = -1.89)*, Carnobacteriaceae* (CLR_diff.btw_ = -2.91, effect size = -0.689)*, Gemellaceae* (CLR_diff.btw_ = -2.45, effect size = -1.31)*,* and *Streptococcaceae* (CLR_diff.btw_ = -2.35, effect size = -1.39)*,* while contain more *Micrococcaceae* (CLR_diff.btw_ = 6.13, effect size = 1.00), *Burkholderiaceae* (CLR_diff.btw_ = 5.24, effect size = 0.913), *Actinomycetaceae* (CLR_diff.btw_ = 4.44, effect size = 1.01), and *Prevotellaceae* (CLR_diff.btw_ = 1.81, effect size = 1.17) as compared to the FL-ONT group (Figure S4D, Table S7C). Importantly, *Burkholderiaceae* and *Micrococcaceae* were only detected by V3V4-Illumina group (Table S7A).

Overall, the bacteria identified by FL-ONT and V3V4-Illumina group were mostly from the same lineage at the phylum, class, order, and family taxonomical levels. However, we also detected bacteria that were unique to the sequencing technique, albeit detected at very low abundance (< 0.1%) (Table S4-S7). Furthermore, we observed decreasing correlation between the relative abundance of FL-ONT and V3V4-Illumina group from higher (phylum) to lower (family) taxonomic groups (Figure 3). Finally, we also observed that there was a good concordance in the relative abundance of the top bacteria detected, whereby both techniques have similar top bacteria detected.

1. **Supplementary Figures:**

**
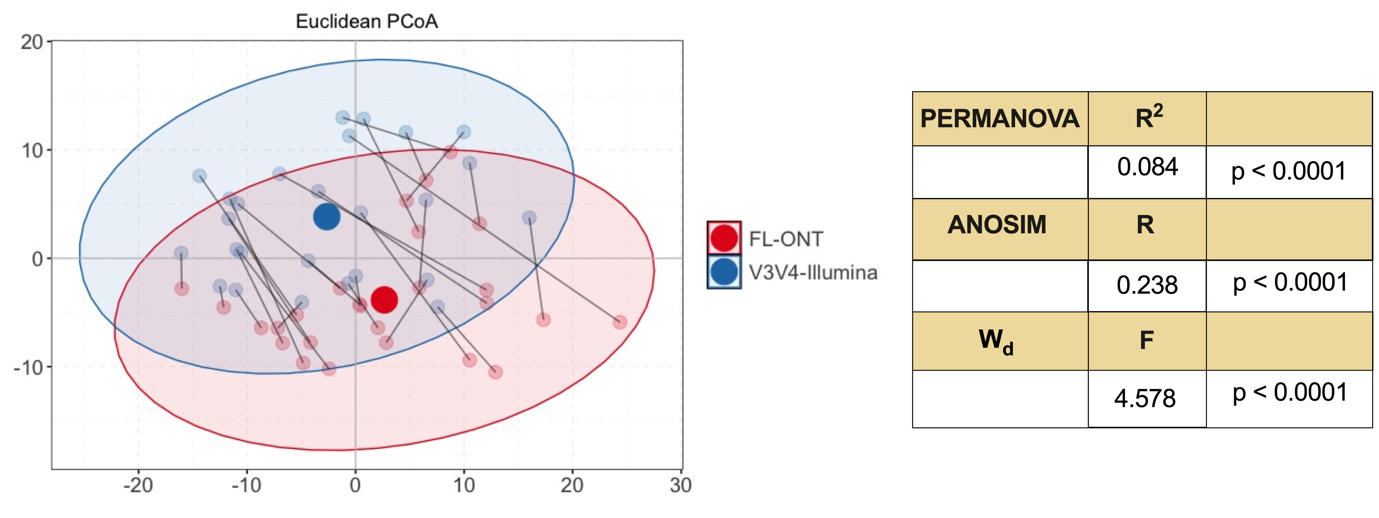
**

**Figure S1: Paired beta diversity analysis of paired FL-ONT and V3V4-Illumina 16S rRNA sequencing on tissue samples at the genus level.** Principal Coordinate Analysis (PCoA) plot of Euclidean distance on CLR normalized abundance. PERMANOVA, ANOSIM and W_d_ test were performed, statistics and p-value were presented. Red and blue dot-points represents ONT and Illumina 16S rRNA sequencing respectively, while line between dot-points represents paired samples.


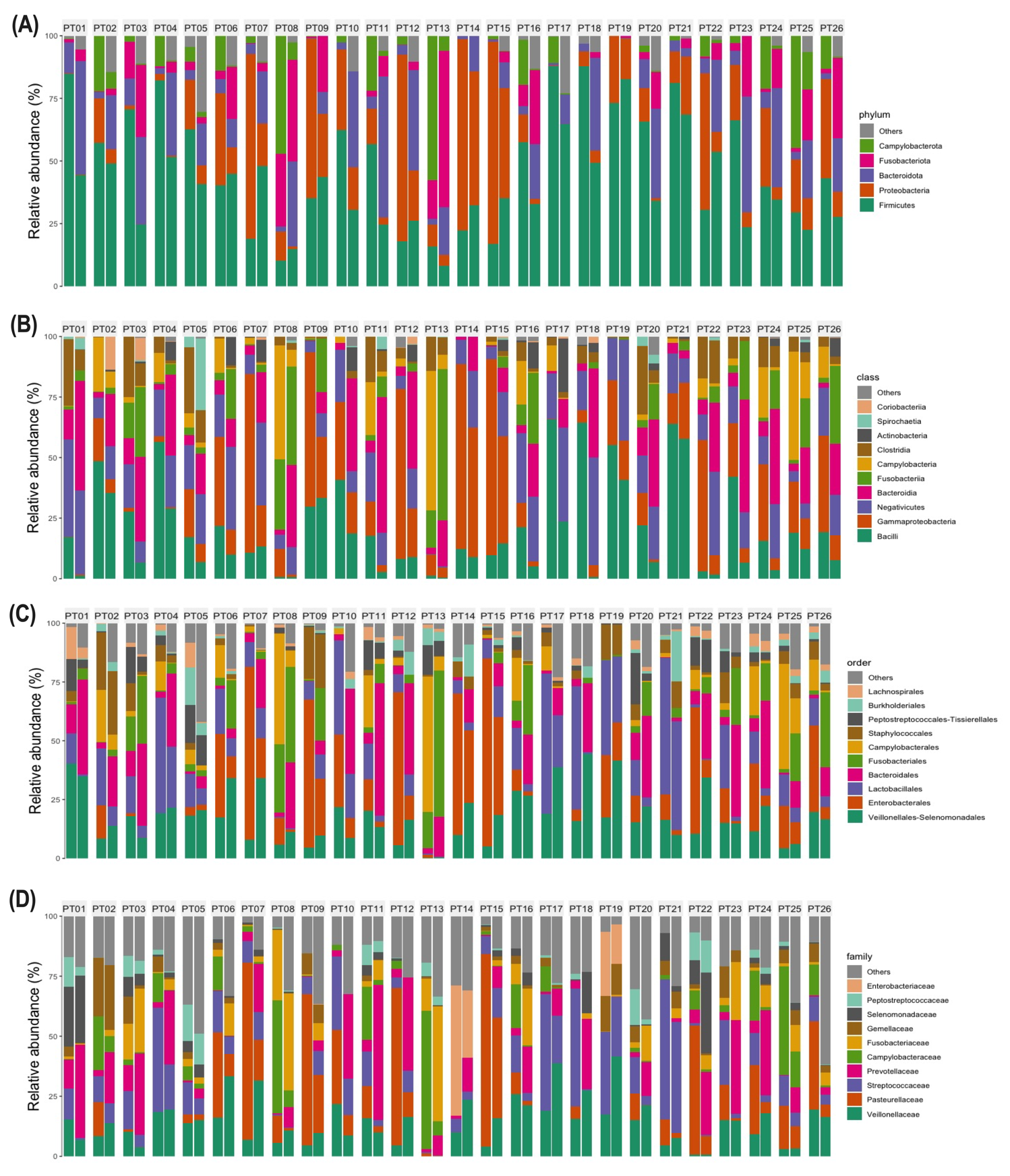


**Figure S2: Relative abundance comparison between FL-ONT and V3V4-Illumina 16S rRNA sequencing from phylum to family levels.** Relative abundance of top (A) Phylum, (B) Class, (C) Order, and (D) Family after agglomerating to each level. For each patient panel (PT01-PT26), the FL-ONT is shown on the left and V3V4-Illumina on the right. Paired Wilcoxon tests were performed to compare differences between FL-ONT to V3V4-Illumina sequencing (Supplementary Table S3-S6).


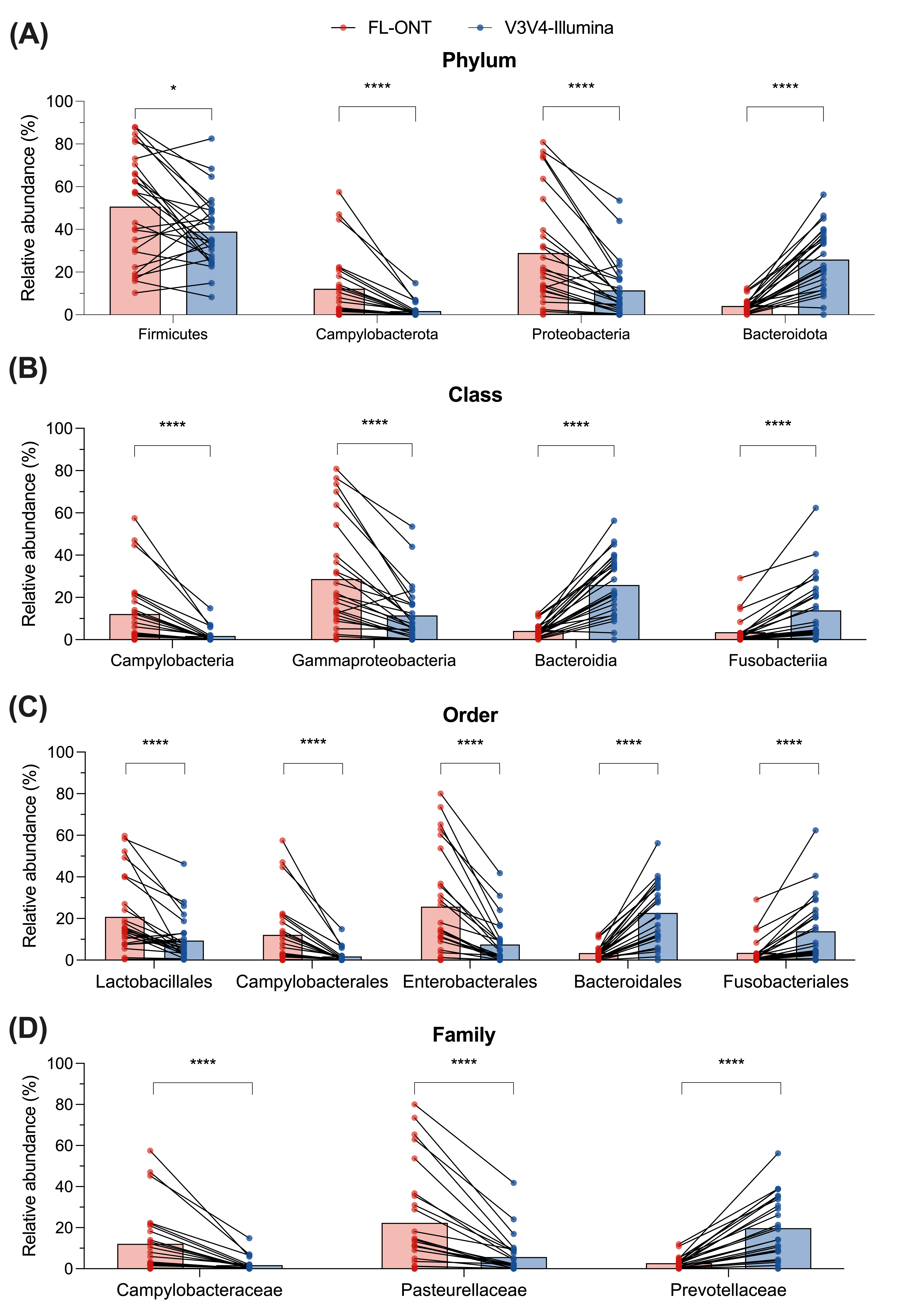


**Figure S3: Comparison of relative abundance between FL-ONT and V3V4-Illumina 16S rRNA sequencing at (A) Phylum, (B), Class, (C) Order and (D) Family levels.** After agglomerating to each taxonomy level, relative abundance was compared between both sequencing techniques (Supplementary Table S4-S7). Relative abundance (%) at each taxonomic levels with > mean 10% differences between techniques. Paired Wilcoxon were performed to compare differences between ONT to Illumina sequencing. ****p < 0.0001, *p < 0.05.


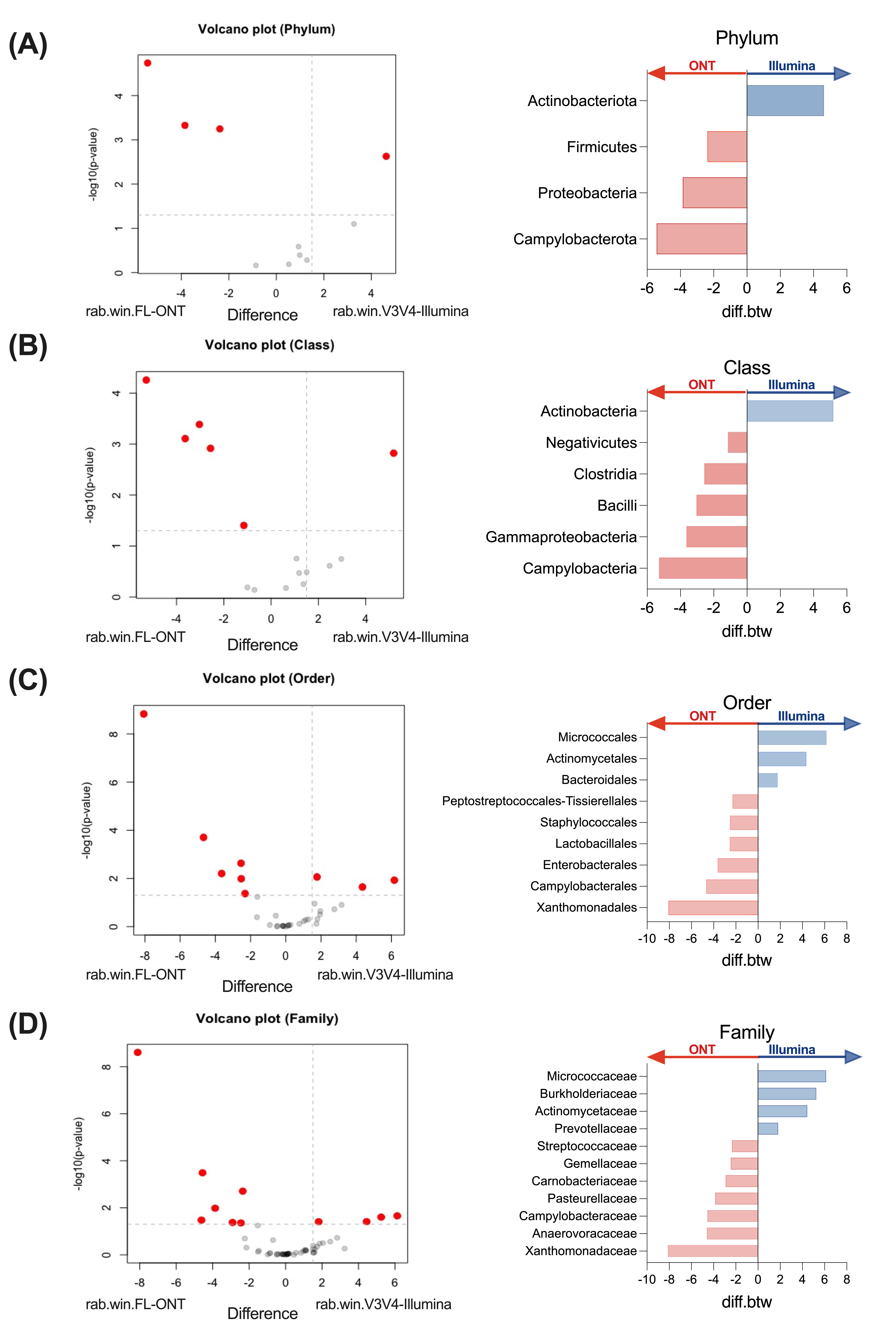


**Figure S4: ALDEx2 analysis show taxonomic differences between FL-ONT and V3V4-Illumina 16S rRNA sequencing.** ALDEx2 analysis was applied at (A) Phylum, (B) Class, (C) Order, and (D) Family levels. Volcano plot (left panel) shows differences in bacteria from phylum to family levels between ONT and Illumina. Red points represent -log_10_(p-value) < 0.05. Points to the left of 0 represent enrichment in Illumina, to the right represent enrichment in ONT. Taxa that were significantly different at all levels were represented in bar plots (right panel). The of bar plot represents bacteria being more abundant in FL-ONT (red) or V3V4-Illumina (blue) sequencing technique.

**References:**

Fernandes AD, Reid JN, Macklaim JM, McMurrough TA, Edgell DR, Gloor GB (2014) Unifying the analysis of high-throughput sequencing datasets: characterizing RNA-seq, 16S rRNA gene sequencing and selective growth experiments by compositional data analysis. Microbiome 2:15 doi: 10.1186/2049-2618-2-15

Hamidi B, Wallace K, Vasu C, Alekseyenko AV (2019) W(∗)(d) -test: robust distance-based multivariate analysis of variance. Microbiome 7:51 doi: 10.1186/s40168-019-0659-9
